# Subclinical Structural and Functional Retinal Alterations in Clinically Normal Myopic Eyes

**DOI:** 10.3390/jcm15135094

**Published:** 2026-06-30

**Authors:** Enes Ozturk, Kuddusi Erkilic

**Affiliations:** 1 Department of Ophthalmology, Kayseri City Hospital, Kayseri 38080, Türkiye; 2 Department of Ophthalmology, School of Medicine, Erciyes University, Kayseri 38039, Türkiye; kuderk@erciyes.edu.tr

**Keywords:** myopia, optical coherence tomography, visual field, axial length, OCT-NFCT, central retinal thickness

## Abstract

**Background/Objectives**: To evaluate subclinical structural and functional retinal alterations in clinically normal myopic eyes using optical coherence tomography (OCT) and automated visual field analysis. **Methods**: This retrospective observational study included 109 patients aged 18–42 years categorized according to spherical refractive component, while spherical equivalent (SE) was calculated to quantify refractive severity. Only the right eye of each participant was analyzed. Patients were categorized into five refractive groups according to spherical refractive component. All participants underwent comprehensive ophthalmologic examination, axial length (AL) measurement, spectral-domain OCT imaging, and standard automated perimetry. Structural parameters included central retinal thickness (CRT) and OCT-derived nerve fiber layer central thickness (OCT-NFCT). Functional assessment included visual field central zone sensitivity (VF-CZ), peripheral zone defect value (VF-PZ), mean deviation/defect value (VF-MD), and square root of loss variance (VF-sLV). Correlation and regression analyses were performed to evaluate relationships among absolute myopia severity, AL, OCT parameters, and visual field indices. **Results**: Increasing myopia was associated with significant reductions in CRT (*p* < 0.001) and significant intergroup differences in OCT-NFCT (*p* = 0.012). A moderate negative correlation was observed between absolute spherical equivalent/myopia severity and CRT (r = −0.5398, *p* < 0.001), whereas the association with OCT-NFCT was not statistically significant (*p* = 0.149). Visual field parameters demonstrated significant deterioration with increasing myopia (all *p* < 0.001). VF-CZ showed a strong negative correlation with myopia severity (r = −0.8545, *p* < 0.001), whereas VF-PZ (r = 0.8196, *p* < 0.001), VF-MD (r = 0.8186, *p* < 0.001), and VF-sLV (r = 0.7606, *p* < 0.001) demonstrated strong positive correlations. In multivariable regression analysis, BCVA, AL, and absolute SE were independently associated with VF-CZ, whereas age and absolute SE were independently associated with VF-MD. CRT and OCT-NFCT were not independent predictors of visual field outcomes. **Conclusions**: Clinically normal myopic eyes demonstrate significant subclinical structural and functional retinal alterations. In this cross-sectional dataset, visual field alterations were detectable across refractive categories and appeared to be more strongly associated with axial elongation and myopia severity than isolated retinal thickness alterations. Combined OCT and visual field assessment may be valuable for early detection and monitoring of subclinical retinal involvement in myopic patients.

## 1. Introduction

Myopia is one of the most common ocular disorders worldwide and has emerged as a major global public health concern due to its rapidly increasing prevalence and associated risk of irreversible visual impairment [1,2,3]. Traditionally regarded as a refractive error resulting from axial elongation of the globe, myopia is now increasingly recognized as a progressive neurostructural ocular condition characterized by biomechanical, vascular, and retinal alterations that may occur even before clinically visible degenerative fundus changes develop. Excessive axial elongation may induce retinal stretching, choroidal thinning, photoreceptor displacement, and mechanical stress on inner retinal structures, potentially leading to subtle impairment of retinal integrity and function.

Although visual acuity may remain relatively preserved in many myopic individuals, increasing evidence suggests that retinal dysfunction can occur at subclinical stages. Previous studies have demonstrated alterations in contrast sensitivity, retinal sensitivity, and visual field performance in myopic eyes, suggesting that myopia may influence retinal physiology beyond refractive blur alone [4,5]. Importantly, these functional abnormalities may be detectable even when overt retinal degeneration is not clinically apparent. Therefore, conventional ophthalmologic examination alone may underestimate early retinal involvement in myopia.

Optical coherence tomography (OCT) has become an essential imaging modality for evaluating retinal microstructure in vivo. High-resolution OCT imaging enables quantitative assessment of retinal thickness and retinal nerve fiber layer (RNFL) integrity, allowing detection of subtle retinal alterations that may occur in the absence of clinically apparent pathology [6]. Several studies have demonstrated that increasing myopia and axial elongation are associated with retinal and RNFL thinning; however, the clinical significance and functional correlates of these structural alterations remain incompletely understood [7,8,9,10]. Furthermore, previous investigations have largely focused either on structural retinal parameters or on functional visual field assessment separately, while the relationship between structural retinal changes and functional retinal sensitivity in clinically normal myopic eyes remains insufficiently characterized.

Visual field testing provides a quantitative evaluation of retinal function and may reveal early neuroretinal dysfunction even in eyes without apparent structural degeneration. Importantly, structural and functional alterations do not necessarily progress in parallel, and functional abnormalities may be detectable even when overt structural loss is not clinically apparent in certain ocular conditions [11,12]. Understanding the interaction between retinal structure and function in myopia may improve early detection of subclinical retinal involvement and contribute to better risk stratification in myopic patients.

Therefore, the present study aimed to investigate whether clinically normal myopic eyes demonstrate evidence of subclinical retinal involvement through combined structural and functional retinal assessment. Using OCT-derived retinal thickness measurements together with automated visual field analysis, we evaluated the relationship between increasing degrees of myopia and neuroretinal alterations in patients without clinically detectable degenerative fundus findings.

## 2. Materials and Methods

### 2.1. Study Design and Ethical Approval

This retrospective observational study was conducted at the Department of Ophthalmology, Erciyes University Medical Faculty Hospital, between June 2019 and March 2021. The study protocol was approved by the Erciyes University Clinical Research Ethics Committee (Approval No: 2022/206; Approval Date: 9 February 2022) and adhered to the tenets of the Declaration of Helsinki.

### 2.2. Participants

A total of 109 patients aged 18–42 years with refractive status classified according to spherical equivalent (SE) were included in the study. The cohort comprised 4 eyes with simple myopia, 100 eyes with myopic astigmatism, and 5 eyes with astigmatism without a spherical myopic component; these 5 astigmatism-only eyes were all within the Group 1 emmetropic-to-very-low-myopia reference range. No astigmatism-only eye was included in Groups 2–5. The astigmatic component ranged from 0.25 to 4.00 D. To avoid inter-eye correlation bias, only the right eye of each participant was analyzed. All participants had a best-corrected visual acuity (BCVA) of 0.7 or higher (Snellen equivalent). No included eye had clinically suspicious glaucomatous optic neuropathy, ocular hypertension, antiglaucoma medication use, or repeatable glaucomatous visual field defects based on the available clinical records.

### 2.3. Inclusion and Exclusion Criteria

Patients with varying degrees of myopia without clinically detectable degenerative retinal pathology were included. Exclusion criteria comprised any ocular or systemic condition that could affect retinal structure or visual field sensitivity, including cataract, corneal opacity, corneal ectatic disorders, glaucoma, uveitis, vitreous opacities, hereditary or acquired retinal diseases, diabetes mellitus, and hypertension. In addition, eyes with intraocular pressure outside the normal range (>21 mmHg), a history of glaucoma or antiglaucoma medication use, suspicious glaucomatous optic neuropathy, focal neuroretinal rim thinning or notching, optic disk hemorrhage, marked cup-to-disk asymmetry, or repeatable glaucomatous visual field defects were excluded. Available records were also reviewed for glaucoma-related history, including documented family history when available.

All participants underwent comprehensive ophthalmologic examination, including best-corrected visual acuity assessment, slit-lamp biomicroscopy, intraocular pressure measurement, dilated fundus examination, refractive error analysis, axial length measurement, OCT imaging, and automated visual field testing. Refractive errors were recorded as spherical equivalent values.

### 2.4. Grouping of Patients

Patients were categorized into five refractive severity groups according to the spherical refractive component recorded in the clinical refraction data, while spherical equivalent (SE) was calculated as spherical refraction plus one-half of the astigmatic refraction and used to quantify refractive severity in descriptive and correlation analyses. Group 1 included eyes with a spherical refractive component between 0 and −0.50 D and was considered an emmetropic-to-very-low-myopia reference group rather than a true myopic group. Because mild astigmatism was present in some Group 1 eyes, the observed absolute SE values in this group ranged from 0.125 to 0.75 D. Groups 2 to 5 represented increasing categories of myopia according to the spherical component: Group 2, −1 to −3 D; Group 3, >−3 to −6 D; Group 4, >−6 to −9 D; and Group 5, ≤−9 D. For descriptive clarity, refractive subtypes were defined as follows: simple myopia indicated a spherical myopic component without astigmatism; myopic astigmatism indicated a spherical myopic component combined with an astigmatic component; and astigmatism-only indicated astigmatism without a spherical myopic component. These refractive subtypes were reported descriptively, whereas the primary analytical groups were based on spherical-component-defined refractive severity, and absolute SE was used for correlation analyses.

### 2.5. Axial Length Measurement

Axial length measurements were obtained using optical biometry with a Nidek biometry system (NIDEK Co., Ltd., Gamagori, Japan). Measurements were performed under standardized conditions by experienced technicians. For each eye, 10 consecutive axial length readings were obtained, and the mean of reliable measurements was recorded for analysis. Measurements with poor signal quality, poor fixation, or obvious acquisition error were repeated or excluded from averaging. Mean axial length values were then included in correlation and regression analyses with retinal structural and functional parameters.

### 2.6. Optical Coherence Tomography Analysis

Retinal imaging was performed using spectral-domain OCT (Spectralis OCT, Heidelberg Engineering, Heidelberg, Germany). Macular volume scans centered on the fovea were used for macular retinal layer analysis. High-quality scans with adequate centration and image quality were included in the analysis. Scan quality and segmentation outputs were reviewed before analysis, and eyes with poor signal quality, motion artifacts, decentration, or obvious segmentation errors were excluded. Manual correction of segmentation errors was not performed; scans with clear segmentation failure were excluded instead. OCT measurements were analyzed using the device-derived automated outputs, and no additional axial length-related magnification correction was applied.

Macular retinal thickness measurements were obtained using the device automated segmentation software. Central retinal thickness (CRT) and OCT-derived nerve fiber layer central thickness (OCT-NFCT) values were recorded for analysis. The OCT-NFCT parameter used in this study did not represent conventional average or sectoral peripapillary RNFL thickness. Instead, OCT-NFCT referred to the device-derived nerve fiber layer central thickness measured within the central macular analysis region by automated segmentation. Values were recorded in micrometers (µm). Segmentation outputs were reviewed for acquisition quality and anatomical consistency prior to statistical evaluation. Quantitative OCT parameters were compared among myopia groups to investigate structural retinal alterations associated with increasing myopia severity and axial elongation.

### 2.7. Visual Field Testing

Visual field assessment was performed using the OCTOPUS 900 perimeter (Haag-Streit Diagnostic International, Koeniz, Switzerland) with the 30–2 threshold test program using the Dynamic strategy and a Goldmann size III stimulus under standard background luminance conditions (31.4 apostilbs). Standard automated threshold perimetry was performed under appropriate refractive correction, and trial lens correction was selected according to the participant’s refractive error. Testing was performed under physiological non-mydriatic pupil conditions, and fixation stability was monitored during the examination. At least two visual field examinations were obtained at separate visits to minimize learning effects and to assess repeatability; the most reliable and repeatable examination was included in the final analysis. Tests were excluded if fixation losses exceeded 20%, false-positive responses exceeded 33%, or false-negative responses exceeded 33%, or if fixation instability, poor cooperation, media opacity, optical defocus, inadequate refractive correction, or lens rim artifact was suspected. Visual field values were recorded in decibels (dB).

The following visual field parameters were evaluated: visual field central zone sensitivity (VF-CZ), visual field peripheral zone defect value (VF-PZ), visual field mean deviation/defect value (VF-MD), and square root of loss variance (VF-sLV). The central zone was defined using the central test locations of the 30–2 grid, whereas the peripheral zone was defined using the remaining peripheral test locations. VF-CZ represents central sensitivity; therefore, lower VF-CZ values indicate worse central visual field function. VF-PZ represents a peripheral zone defect/depression value in this dataset; therefore, higher VF-PZ values indicate worse peripheral visual field function. Similarly, higher VF-MD and VF-sLV values indicate greater diffuse and localized visual field impairment, respectively. These parameters were compared among myopia groups and correlated with SE, axial length, and OCT-derived retinal measurements.

### 2.8. Statistical Analysis

Statistical analyses were performed using TURCOSA software (Turcosa Analytics Co., Ltd., Kayseri, Turkey; www.turcosa.com.tr, accessed on 26 June 2026). Normality of continuous variables was assessed using the Shapiro–Wilk test. According to normality testing, most primary quantitative variables did not meet normal distribution assumptions; therefore, data were generally presented as median (interquartile range), while normally distributed variables were presented as mean ± standard deviation. For comparisons among the five refractive groups, one-way analysis of variance (ANOVA) with Tukey post hoc testing was used for normally distributed variables, whereas the Kruskal–Wallis test with Dunn-Bonferroni post hoc correction was used for non-normally distributed variables. Categorical variables were compared using the chi-square test or Fisher’s exact test, as appropriate. Post hoc *p*-values were multiple-comparison-adjusted. Correlation analyses were performed using Spearman’s rank correlation coefficient because the main refractive, OCT, and visual field variables were predominantly non-normally distributed. Linear regression analyses were used to evaluate potential predictors of visual field outcomes. Variables with *p* < 0.05 in univariable regression analyses were entered into multivariable regression models. Multicollinearity in multivariable regression models was assessed using the variance inflation factor (VIF), and VIF values below 5 were considered acceptable. A two-sided *p*-value of <0.05 was considered statistically significant.

Because this was a retrospective study based on available clinical records, an a priori sample size calculation was not performed before data collection. However, a post hoc minimum sample size calculation for a five-group comparison using a one-way ANOVA framework, alpha = 0.05, power = 0.80, and a conservative large effect size (Cohen f = 0.40) indicated a minimum total sample size of approximately 80 eyes. The present study included 109 eyes, with 21–23 eyes in each refractive category, exceeding this estimated minimum sample size for detecting large intergroup effects.

Because age differed significantly among the refractive groups, age-adjusted analyses were performed for the main structural and functional outcome variables. Analysis of covariance (ANCOVA) was used to evaluate group differences after adjustment for age, with group as the fixed factor and age as the covariate. Age-adjusted results were reported alongside the unadjusted group comparisons where appropriate.

For correlation analyses, spherical equivalent was coded as the absolute magnitude of myopia severity rather than as signed negative dioptric values; therefore, higher SE values indicate greater myopia severity. For example, an eye with −6.00 D of spherical equivalent was analyzed as 6.00 D in correlation analyses.

## 3. Results

A total of 109 patients (54 males [49.5%] and 55 females [50.5%]) were included in the study, and only right eyes were analyzed. The participants were categorized into five groups according to the spherical refractive component recorded in the clinical refraction data. Group 1 was defined as an emmetropic-to-very-low-myopia reference group, whereas Groups 2 to 5 represented increasing categories of myopia severity. Group sizes were as follows: Group 1, *n* = 22; Group 2, *n* = 21; Group 3, *n* = 22; Group 4, *n* = 21; and Group 5, *n* = 23. Descriptively, 4 eyes had simple myopia, 100 eyes had myopic astigmatism, and 5 eyes had astigmatism-only within the Group 1 reference range. Therefore, the five analytical groups were based on spherical-component-defined refractive severity, while absolute SE values and the simple myopia/myopic astigmatism/astigmatism-only categories were reported descriptively and used where appropriate for correlation analyses.

Intraocular pressure and optic disk parameters were within normal limits in all included eyes. The mean intraocular pressure was 14.3 ± 2.1 mmHg, ranging from 10.2 to 18.7 mmHg. The mean vertical cup-to-disk ratio was 0.29 ± 0.06, ranging from 0.18 to 0.44, and the mean horizontal cup-to-disk ratio was 0.28 ± 0.06, ranging from 0.16 to 0.44. No eye had ocular hypertension, suspicious glaucomatous optic neuropathy, or a repeatable glaucomatous visual field defect.

Age distribution differed significantly among the groups (*p* = 0.006). Spherical equivalent values showed significant differences among all groups (*p* < 0.001). BCVA values decreased significantly with increasing myopia (*p* < 0.001) (Table 1). Because age differed significantly among groups, age-adjusted analyses were performed for the main structural and functional outcomes.

Axial length values increased significantly with increasing myopia (*p* < 0.001). Median AL values were 24.2 (23.7–24.5) mm in Group 1, 24.4 (23.9–24.9) mm in Group 2, 25.2 (24.5–25.8) mm in Group 3, 26.5 (25.7–27.1) mm in Group 4, and 28.9 (28.1–29.6) mm in Group 5 (Table 1). Post hoc analysis demonstrated significant differences between Groups 1 and 3 (*p* = 0.008), Groups 1 and 4 (*p* < 0.001), Groups 1 and 5 (*p* < 0.001), Groups 2 and 4 (*p* = 0.007), Groups 2 and 5 (*p* < 0.001), and Groups 3 and 5 (*p* = 0.008). A strong positive correlation was observed between the absolute magnitude of SE/myopia severity and AL values (r = 0.813, *p* < 0.001).Visual field parameters demonstrated significant differences among the groups (all *p* < 0.001). Median VF-CZ values progressively decreased from 25.0 (24.0–26.0) in Group 1 to 12.0 (9.5–13.0) in Group 5, indicating reduced central visual field sensitivity. Similarly, VF-PZ defect values increased from 1.7 (1.4–2.1) in Group 1 to 9.5 (8.6–11.6) in Group 5. VF-MD values also increased progressively with increasing myopia. In this dataset, higher VF-PZ, VF-MD, and VF-sLV values indicate worse visual field function (Table 2).

Correlation analysis was performed using SE coded as the absolute magnitude of myopia severity. Absolute SE/myopia severity showed a moderate negative correlation with CRT (r = −0.5398, *p* < 0.001), whereas the correlation with OCT-NFCT did not reach statistical significance (r = −0.1885, *p* = 0.149). Strong negative correlations were observed between absolute SE/myopia severity and VF-CZ (r = −0.8545, *p* < 0.001). In contrast, strong positive correlations were identified between absolute SE/myopia severity and VF-PZ (r = 0.8196, *p* < 0.001), VF-MD (r = 0.8186, *p* < 0.001), and VF-sLV (r = 0.7606, *p* < 0.001) (Table 3).

OCT parameters also differed significantly among the groups. Median CRT values were 269.0 (257.0–280.3) µm in Group 1, 272.0 (257.8–283.0) µm in Group 2, 261.0 (244.3–276.5) µm in Group 3, 247.0 (237.0–267.8) µm in Group 4, and 220.0 (198.0–247.3) µm in Group 5, demonstrating progressive retinal thinning with increasing myopia (*p* < 0.001). OCT-NFCT values also showed significant intergroup differences (*p* = 0.012) (Table 4).

Post hoc intergroup analysis demonstrated that statistically significant visual field alterations were observed at lower degrees of myopia compared with some OCT-derived structural parameters. Significant differences for VF-CZ, VF-PZ, VF-MD, and VF-sLV were detected beginning from comparisons involving Groups 1 and 4, whereas CRT and OCT-NFCT differences became more prominent mainly in comparisons involving Group 5 (Table 5). In this cross-sectional dataset, visual field alterations were detectable in lower-myopia categories than some OCT-derived structural parameters; however, the temporal sequence of functional and structural changes cannot be determined.

Univariable regression analyses were performed to evaluate potential predictors of visual field outcomes, including age, BCVA, AL, SE, CRT, OCT-NFCT, and sex. Age, BCVA, AL, and SE were significantly associated with both VF-CZ and VF-MD. CRT, OCT-NFCT, and sex were not significantly associated with either VF-CZ or VF-MD in univariable analyses, although OCT-NFCT showed a borderline but non-significant association with VF-CZ (β = 0.212, *p* = 0.079).

Variables that were statistically significant in univariable analyses were included in the multivariable regression models. In the final model for VF-CZ, BCVA (β = 13.726, *p* < 0.001), AL (β = −0.570, *p* = 0.001), and SE (β = −0.859, *p* < 0.001) remained independent predictors, whereas age was no longer statistically significant. In the final model for VF-MD, age (β = 0.047, *p* = 0.003) and SE (β = 0.539, *p* < 0.001) remained significant independent predictors, whereas BCVA and AL were not independently associated with VF-MD after adjustment (Table 6). These findings indicate that visual field impairment is influenced by refractive severity and selected optical/functional factors and therefore should not be interpreted solely as an isolated effect of axial elongation. Although SE and AL were strongly related, multicollinearity assessment demonstrated no severe multicollinearity in the final models, with all VIF values below 5 and a maximum VIF of 3.31.

**Figure 1:**
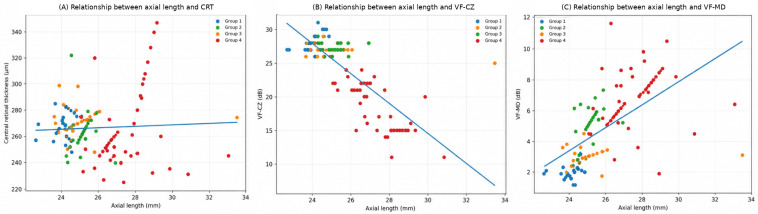
Relationship between axial length and key structural and functional parameters. (**A**) shows the relationship between axial length (AL) and central retinal thickness (CRT). (**B**) shows the relationship between AL and visual field central zone sensitivity (VF-CZ). (**C**) shows the relationship between AL and visual field mean deviation/defect value (VF-MD). The blue line represents the fitted linear regression line.

To improve readability and to visually summarize the relationship between axial length and key structural and functional parameters were presented in Figure 1.

## 4. Discussion

The present study evaluated both structural and functional retinal changes in myopic patients without clinically detectable degenerative fundus findings by integrating OCT segmentation analysis and visual field sensitivity measurements. The findings demonstrate that increasing myopia is associated with significant alterations in retinal thickness parameters as well as measurable reductions in visual field sensitivity, even in the absence of overt pathological changes.

Myopia is known to be associated with axial elongation and biomechanical stretching of ocular tissues, which may lead to progressive thinning of retinal layers. Previous studies have shown that even in non-pathological myopia, subtle structural changes may occur in the retina and RNFL, which may occur before or without clinically detectable degeneration [9,10]. In addition to previous studies, our research evaluated OCT-NFCT and visual field test data together, integrating both structural and functional outcomes across patient groups. This combined assessment may provide valuable insight into the potential role of integrated diagnostic modalities in clinical follow-up.

In line with these findings, our study demonstrated a significant decrease in CRT with increasing myopia, accompanied by a moderate-to-strong negative correlation with absolute SE/myopia severity. These findings support the concept that axial elongation-related retinal stretching leads to measurable structural alterations. Similarly, OCT-NFCT showed a decreasing trend with increasing myopia. However, although a negative correlation was observed, this relationship did not reach statistical significance. This finding may suggest that OCT-derived nerve fiber layer central thickness changes in early or moderate myopia are less pronounced or more variable compared with total retinal thickness. Previous studies using ocular imaging modalities have reported conflicting results regarding nerve fiber layer measurements in myopia, possibly depending on axial length, measurement region, and imaging/acquisition protocol [13,14,15].

Several mechanisms have been proposed to explain nerve fiber layer alterations in myopic eyes. Myopia leads to elongation of the axial length of the eye, resulting in mechanical stretching and subsequent thinning of retinal neural layers. In addition, myopic retinal degeneration may further contribute to nerve fiber layer changes. Furthermore, increased axial length may affect the sensitivity and measurement accuracy of OCT devices, potentially leading to artifacts in nerve fiber layer thickness assessment.

In addition to structural changes, our study revealed significant functional impairment in visual field parameters. VF-CZ showed a strong negative correlation with increasing myopia, whereas VF-PZ demonstrated a strong positive correlation. These findings indicate that retinal sensitivity decreases centrally while peripheral visual field defects become more prominent as myopia progresses. Notably, these alterations were observed despite the absence of clinically detectable retinal pathology, highlighting the presence of subclinical functional impairment.

Global visual field indices such as MD and sLV demonstrated strong positive correlations with myopia severity. Increased MD values reflect generalized depression of retinal sensitivity, while elevated sLV values indicate increased variability and localized defects. These findings suggest that myopia is associated with both diffuse and focal functional impairment in the visual field. The relationship between structural and functional findings is particularly important. The observed reduction in retinal thickness alongside decreased visual field sensitivity supports the hypothesis that structural alterations in retinal layers may underlie functional deficits. OCT provides quantitative assessment of retinal morphology, while perimetry reflects functional output of the visual pathway; therefore, combining these modalities offers a comprehensive evaluation of retinal integrity.

In the present study, multivariable regression analysis showed that AL remained independently associated with VF-CZ, whereas absolute SE remained independently associated with both VF-CZ and VF-MD. In contrast, CRT and OCT-NFCT were not independent predictors of visual field outcomes after adjustment. These findings suggest that visual field impairment in myopic eyes is influenced by refractive severity, axial elongation, and selected optical/functional factors, and should not be interpreted solely as an isolated effect of retinal thickness alterations. Axial elongation and increasing refractive severity may be associated with diffuse neuroretinal stress and retinal sensitivity changes even when measurable OCT-derived thinning is not prominent. This observation further supports the concept that visual field alterations may be detectable even when OCT-derived structural changes are less pronounced in clinically normal myopic eyes.

Several studies have demonstrated that increased axial length, one of the primary underlying mechanisms of myopia, may also occur in glaucomatous eyes. Moreover, previous reports have shown that in eyes with clinical or subclinical glaucoma, increased axial length is associated with a higher prevalence of artifacts and alterations in both visual field and OCT measurements [16]. It is important to determine whether detected defects are attributable to glaucoma or represent a consequence of increased axial length. The findings of our study suggest that longitudinal follow-up of early visual field and OCT parameters may aid in the clinical differentiation of pathologies such as glaucoma over time.

Interestingly, the results of this study suggest that functional impairment may be detectable even in the absence of marked structural loss in certain parameters such as OCT-NFCT. This finding may indicate that functional testing, particularly visual field analysis, may provide complementary functional information regarding retinal involvement in myopia. Previous studies have suggested that structural and functional changes may not always occur in parallel in certain ocular conditions [12,17].

In addition, the observation that visual field abnormalities were detectable in lower-myopia categories than some OCT-derived structural parameters suggests potential clinical value in combined functional and structural follow-up. Because the present study was cross-sectional, these findings should not be interpreted as evidence that functional impairment temporally precedes structural change.

This study has several limitations that should be acknowledged. First, the retrospective design may introduce selection bias and limit the ability to establish causal relationships. Second, the sample size, although adequate, may not fully represent all subgroups of myopia, particularly extreme high myopia. Third, although visual field testing was carefully controlled, perimetry is inherently subjective and may be influenced by patient cooperation, fatigue, learning effects, fixation stability, optical correction, pupil size, and magnification-related factors, particularly in high myopia. Although repeated visual field examinations and reliability criteria were used to minimize these effects, residual optical or test-related confounding cannot be completely excluded. Fourth, BCVA was recorded and analyzed as decimal Snellen visual acuity values; although this reflects the clinical data format available in the retrospective records, logMAR transformation would provide a more appropriate scale for statistical modeling and should be considered in future prospective analyses. In particular, the marked reduction in visual field sensitivity observed in the highest myopia group should be interpreted cautiously, as high-myopia-related optical factors such as trial lens effects, lens rim artifact, reduced BCVA, pupil-related factors, and optical defocus may contribute to apparent field loss despite reliability screening. In addition, because no additional axial length-related OCT magnification correction was applied, apparent differences in CRT or OCT-NFCT may partly reflect ocular magnification or scaling artifacts, especially in eyes with longer axial length. Finally, although intraocular pressure and cup-to-disk ratio values were within normal limits in all included eyes, optic disk evaluation was based on retrospective clinical records rather than standardized prospective optic disk photography.

## 5. Conclusions

Increasing myopia is associated with significant structural and functional retinal changes, even in the absence of clinically detectable degenerative findings. Central retinal thickness decreases with higher myopia, while visual field sensitivity shows a marked decline in both central and peripheral regions. The associations between myopia severity and visual field indices suggest that functional alterations may be detectable even when OCT-derived structural changes are less pronounced; however, longitudinal studies are required to determine temporal sequence. These findings highlight the importance of combined OCT and visual field assessment in the early detection and monitoring of subclinical retinal involvement in myopic patients.

## Figures and Tables

**Table 1:** Baseline demographic and clinical characteristics of the study groups.

Variable	Group 1	Group 2	Group 3	Group 4	Group 5	*p*-Value
*n*	22	21	22	21	23	—
Sex, female/male	11/11	11/10	11/11	10/11	12/11	0.998
Age (years)	24.5 (18.0–39.0)	27.5 (21.3–40.5)	29.5 (23.3–37.5)	26.5 (18.0–38.4)	30.5 (23.0–42.0)	0.006
Absolute SE (D)	0.62 (0.28–0.75)	2.88 (2.50–3.25)	4.75 (4.16–5.12)	7.88 (7.50–8.25)	10.88 (10.50–11.38)	<0.001
Astigmatic refraction (D)	0.50 (0.25–0.50)	0.50 (0.25–1.25)	0.75 (0.56–1.25)	1.25 (1.00–1.50)	2.25 (2.00–2.62)	<0.001
BCVA	1.0 (1.0–1.0)	1.0 (1.0–1.0)	1.0 (0.9–1.0)	0.9 (0.9–1.0)	0.8 (0.8–0.8)	<0.001
AL (mm)	24.2 (23.7–24.5)	24.4 (23.9–24.9)	25.2 (24.5–25.8)	26.5 (25.7–27.1)	28.9 (28.1–29.6)	<0.001
IOP (mmHg)	13.8 (12.6–15.1)	14.6 (11.9–16.1)	14.2 (12.2–15.3)	14.7 (13.3–15.7)	15.1 (13.3–16.9)	0.262
Vertical C/D ratio	0.28 (0.24–0.31)	0.28 (0.25–0.30)	0.29 (0.25–0.33)	0.31 (0.26–0.35)	0.31 (0.29–0.37)	0.064

Values are presented as median (interquartile range) unless otherwise indicated. SE: spherical equivalent; BCVA: best-corrected visual acuity; AL: axial length. Groups were defined by spherical component: G1 0 to −0.50 D, G2 −1 to −3 D, G3 −3 to −6 D, G4 −6 to −9 D, and G5 ≤ −9 D. Absolute SE = |sphere + 1/2 cylinder|; therefore, Group 1 absolute SE values ranged from 0.125 to 0.75 D because of mild astigmatism. IOP: intraocular pressure; C/D: cup-to-disk ratio.

**Table 2:** Comparison of visual field parameters among groups.

**Variable**	**Group 1**	**Group 2**	**Group 3**	**Group 4**	**Group 5**	***p*** **-Value**
VF-CZ (dB)	25.0 (24.0–26.0)	24.0 (24.0–25.0)	24.0 (24.0–25.0)	17.5 (17.0–18.8)	12.0 (9.5–13.0)	<0.001
VF-PZ (dB)	1.7 (1.4–2.1)	1.9 (1.6–2.5)	2.8 (1.8–4.1)	5.4 (4.4–7.6)	9.5 (8.6–11.6)	<0.001
VF-MD (dB)	1.3 (1.2–1.6)	1.9 (1.3–2.4)	3.3 (2.5–4.5)	3.6 (2.6–4.4)	6.8 (4.5–8.4)	<0.001
VF-sLV (dB)	1.2 (0.9–2.4)	2.11 (1.10–3.40)	4.10 (2.50–5.40)	3.54 (1.50–6.30)	3.98 (2.20–6.60)	<0.001

VF-CZ: visual field central zone sensitivity; VF-PZ: visual field peripheral zone defect value; VF-MD: visual field mean deviation/defect value; VF-sLV: square root of loss variance. Lower VF-CZ values indicate worse central sensitivity, whereas higher VF-PZ, VF-MD, and VF-sLV values indicate worse visual field function in this dataset.

**Table 3:** Correlation analysis between absolute spherical equivalent/myopia severity and structural and functional parameters.

Variables	Spearman Correlation Coefficient (r)	*p*-Value
Absolute SE vs. AL	0.813	<0.001
Absolute SE vs. CRT	−0.5398	<0.001
Absolute SE vs. OCT-NFCT	−0.1885	0.149
Absolute SE vs. VF-CZ	−0.8545	<0.001
Absolute SE vs. VF-PZ	0.8196	<0.001
Absolute SE vs. VF-MD	0.8186	<0.001
Absolute SE vs. VF-sLV	0.7606	<0.001

SE: spherical equivalent; AL: axial length; CRT: central retinal thickness; OCT-NFCT: OCT-derived nerve fiber layer central thickness. For correlation analyses, SE was coded as the absolute magnitude of myopia severity rather than as signed negative dioptric values; therefore, higher values indicate greater myopia severity.

**Table 4:** Comparison of OCT parameters among groups.

Variable	Group 1	Group 2	Group 3	Group 4	Group 5	*p*-Value
CRT (µm)	269.0 (257.0–280.3)	272.0 (257.8–283.0)	261.0 (244.3–276.5)	247.0 (237.0–267.8)	220.0 (198.0–247.3)	<0.001
OCT-NFCT (µm)	12.5 (11.0–15.8)	15.0 (14.0–16.8)	16.0 (14.0–18.8)	13.0 (8.5–15.5)	12.5 (8.5–14.8)	0.012

CRT: central retinal thickness; OCT-NFCT: OCT-derived nerve fiber layer central thickness measured within the central macular analysis region. OCT-NFCT does not represent conventional average or sectoral peripapillary RNFL thickness.

**Table 5:** Significant post hoc intergroup comparisons of structural and functional parameters.

Parameter	G1–G4	G1–G5	G2–G4	G2–G5	G3–G4	G3–G5
CRT	-	*p* = 0.004	-	*p* < 0.001	-	-
OCT-NFCT	-	*p* < 0.001	-	*p* < 0.001	-	*p* = 0.039
VF-CZ	*p* < 0.001	*p* < 0.001	-	*p* < 0.001	*p* = 0.015	*p* < 0.001
VF-PZ	*p* < 0.001	*p* < 0.001	*p* = 0.033	*p* < 0.001	*p* = 0.020	*p* < 0.001
VF-MD	*p* < 0.001	*p* < 0.001	*p* = 0.010	*p* < 0.001	-	*p* < 0.001
VF-sLV	*p* = 0.004	-	*p* = 0.003	*p* < 0.001	-	*p* < 0.001

Only statistically significant post hoc comparisons are shown. Reported *p*-values are multiple-comparison-adjusted *p*-values obtained using the appropriate post hoc procedure. Dashes indicate non-significant adjusted comparisons.

**Table 6:** Univariable and multivariable regression analyses for visual field outcomes.

Dependent Variable	Analysis	Predictor	β Coefficient	*p*-Value
VF-CZ	Univariable	Age	−0.205	<0.001
VF-CZ	Univariable	BCVA	46.590	<0.001
VF-CZ	Univariable	AL	−2.227	<0.001
VF-CZ	Univariable	SE	−1.255	<0.001
VF-CZ	Univariable	CRT	−0.010	0.161
VF-CZ	Univariable	OCT-NFCT	0.212	0.079
VF-CZ	Univariable	Male sex	0.308	0.761
VF-CZ	Multivariable	Age	0.042	0.066
VF-CZ	Multivariable	BCVA	13.726	<0.001
VF-CZ	Multivariable	AL	−0.570	0.001
VF-CZ	Multivariable	SE	−0.859	<0.001
VF-MD	Univariable	Age	0.121	<0.001
VF-MD	Univariable	BCVA	−16.549	<0.001
VF-MD	Univariable	AL	0.753	<0.001
VF-MD	Univariable	SE	0.515	<0.001
VF-MD	Univariable	CRT	0.003	0.419
VF-MD	Univariable	OCT-NFCT	−0.011	0.849
VF-MD	Univariable	Male sex	−0.175	0.707
VF-MD	Multivariable	Age	0.047	0.003
VF-MD	Multivariable	BCVA	0.600	0.790
VF-MD	Multivariable	AL	−0.171	0.128
VF-MD	Multivariable	SE	0.539	<0.001

VF-CZ: visual field central zone sensitivity; VF-MD: visual field mean deviation/defect value; BCVA: best-corrected visual acuity; AL: axial length; SE: spherical equivalent, expressed as the absolute magnitude of myopia severity; CRT: central retinal thickness; OCT-NFCT: OCT-derived nerve fiber layer central thickness. Variables with *p* < 0.05 in univariable regression analyses were entered into the multivariable regression model. OCT-NFCT was evaluated in univariable analyses but was not included in the final multivariable models because it did not reach statistical significance. Multicollinearity was assessed using VIF; all VIF values in the final models were below 5.

## Data Availability

The data supporting the findings of this study are available from the corresponding author upon reasonable request.
